# Averting an Outbreak of SARS-CoV-2 in a University Residence Hall through Wastewater Surveillance

**DOI:** 10.1128/Spectrum.00792-21

**Published:** 2021-10-06

**Authors:** Ryland Corchis-Scott, Qiudi Geng, Rajesh Seth, Rajan Ray, Mohsan Beg, Nihar Biswas, Lynn Charron, Kenneth D. Drouillard, Ramsey D’Souza, Daniel D. Heath, Chris Houser, Felicia Lawal, James McGinlay, Sherri Lynne Menard, Lisa A. Porter, Diane Rawlings, Matthew L. Scholl, K. W. Michael Siu, Yufeng Tong, Christopher G. Weisener, Steven W. Wilhelm, R. Michael L. McKay

**Affiliations:** a Great Lakes Institute for Environmental Research, University of Windsor, Windsor, Ontario, Canada; b Civil and Environmental Engineering, University of Windsor, Windsor, Ontario, Canada; c Student Counselling Centre, University of Windsor, Windsor, Ontario, Canada; d Residence Services, University of Windsor, Windsor, Ontario, Canada; e School of the Environment, University of Windsor, Windsor, Ontario, Canada; f Windsor-Essex County Health Unit, Windsor, Ontario, Canada; g Department of Integrative Biology, University of Windsor, Windsor, Ontario, Canada; h Environmental Health and Safety, University of Windsor, Windsor, Ontario, Canada; i Department of Biomedical Sciences, University of Windsor, Windsor, Ontario, Canada; j Student Health Services, University of Windsor grid.267455.7University of Windsor, Windsor, Ontario, Canada; k Department of Chemistry and Biochemistry, University of Windsor, Windsor, Ontario, Canada; l Department of Microbiology, The University of Tennessee, Knoxville, Tennessee, USA; m Great Lakes Center for Fresh Waters and Human Health, Bowling Green State University, Bowling Green, Ohio, USA; Johns Hopkins Hospital

**Keywords:** COVID-19, RT-qPCR, SARS-CoV-2, wastewater

## Abstract

A wastewater surveillance program targeting a university residence hall was implemented during the spring semester 2021 as a proactive measure to avoid an outbreak of COVID-19 on campus. Over a period of 7 weeks from early February through late March 2021, wastewater originating from the residence hall was collected as grab samples 3 times per week. During this time, there was no detection of SARS-CoV-2 by reverse transcriptase quantitative PCR (RT-qPCR) in the residence hall wastewater stream. Aiming to obtain a sample more representative of the residence hall community, a decision was made to use passive samplers beginning in late March onwards. Adopting a Moore swab approach, SARS-CoV-2 was detected in wastewater samples just 2 days after passive samplers were deployed. These samples also tested positive for the B.1.1.7 (Alpha) variant of concern (VOC) using RT-qPCR. The positive result triggered a public health case-finding response, including a mobile testing unit deployed to the residence hall the following day, with testing of nearly 200 students and staff, which identified two laboratory-confirmed cases of Alpha variant COVID-19. These individuals were relocated to a separate quarantine facility, averting an outbreak on campus. Aggregating wastewater and clinical data, the campus wastewater surveillance program has yielded the first estimates of fecal shedding rates of the Alpha VOC of SARS-CoV-2 in individuals from a nonclinical setting.

**IMPORTANCE** Among early adopters of wastewater monitoring for SARS-CoV-2 have been colleges and universities throughout North America, many of whom are using this approach to monitor congregate living facilities for early evidence of COVID-19 infection as an integral component of campus screening programs. Yet, while there have been numerous examples where wastewater monitoring on a university campus has detected evidence for infection among community members, there are few examples where this monitoring triggered a public health response that may have averted an actual outbreak. This report details a wastewater-testing program targeting a residence hall on a university campus during spring 2021, when there was mounting concern globally over the emergence of SARS-CoV-2 variants of concern, reported to be more transmissible than the wild-type Wuhan strain. In this communication, we present a clear example of how wastewater monitoring resulted in actionable responses by university administration and public health, which averted an outbreak of COVID-19 on a university campus.

## INTRODUCTION

Novel coronavirus disease 2019 (COVID-19) is an acute respiratory disease that first came to the attention of the World Health Organization in early 2020. The pathogen responsible for COVID-19 is SARS-CoV-2, a member of the coronavirus family (1). By the end of 2020, >84 million cases and >1.8 million deaths had been reported worldwide (2). These statistics, however, underestimate the actual levels of infection, as many patients are asymptomatic (3–5) or present with only mild symptoms and do not seek medical attention. Indeed, undocumented infections may explain the initial rapid geographic spread of COVID-19 across the globe (6). Therefore, it is of high priority to public health to optimize and expand appropriate screening and surveillance that can recognize the true prevalence of infection.

Wastewater monitoring offers a promising and cost-effective alternative to the large-scale testing of individuals, as shown by a growing body of research from across the globe that has demonstrated the presence of SARS-CoV-2 RNA in sewage from wastewater treatment plants (WWTPs) (7–9), consistent with studies showing that the novel coronavirus is shed in feces (10, 11). A 24-h composite sample of raw sewage represents the fecal discharge of the entire community served by the WWTP, effectively providing a community-wide swab. Taking into consideration rates of viral shedding, decay of the RNA signal within the sewershed, and sensitivity of the assay, modeling and numerical analysis suggest the potential to detect a single infection in a population from one hundred to 2 million people (12). Thus, data on the SARS-CoV-2 viral load in wastewater can be used to inform municipalities and public health units on trends in community infections in the absence of wide-scale testing of individuals. This is consistent with wastewater-based epidemiology (WBE) programs implemented for pathogens such as the polio virus (13, 14). That SARS-CoV-2 can be shed even before manifestations of COVID-19 become apparent in an infected individual makes this approach even more powerful, especially for early warning (15). Indeed, WBE data from around the globe has identified SARS-CoV-2 RNA in wastewater prior to clinical metrics of infections in the communities served by those WWTPs (9, 16–18).

While informative of higher-level trends in community health (19), relying solely on WWTPs for sampling limits the epidemiological value of wastewater surveillance. Indeed, to yield the most benefit, strategic sampling within a sewershed targeting neighborhoods, schools, or congregate living facilities provides finer spatial resolution that can result in actionable public health responses to limit or halt COVID-19 transmission (20). Among early adopters of this approach have been colleges and universities throughout North America, many of whom are using WBE to monitor residence halls for early evidence of COVID-19 infection as an integral component of campus screening programs (18, 21–30). In fact, by the completion of the 2020 to 2021 academic year, >200 postsecondary institutions in North America and >250 worldwide were involved in some form of wastewater surveillance on campus (31). Yet while there have been numerous examples where wastewater monitoring on a university campus has detected evidence for infection among community members, there are few examples where this monitoring triggered a public health response that may have averted an actual outbreak (22, 26). Notable in this respect was a high-profile case at the University of Arizona in August 2020, where WBE triggered targeted clinical testing of students living in a residence hall that identified 3 individuals (2 of whom were asymptomatic) who subsequently tested positive for COVID-19 (22, 32). The infected students were relocated to a quarantine facility until they were deemed to be no longer infectious.

As part of the Province of Ontario’s Wastewater Surveillance Initiative (33), WBE was established at the University of Windsor, where wastewater originating from a residence hall was monitored thrice-weekly beginning in February 2021. Here, we describe a case study where wastewater surveillance triggered a public health response that potentially averted an outbreak of the Alpha variant of concern (VOC) on a university campus.

## RESULTS AND DISCUSSION

### Campus wastewater surveillance for SARS-CoV-2.

A wastewater surveillance program on the campus of the University of Windsor was initiated in early February 2021, near the end of a provincial lockdown as the Windsor-Essex County region was emerging from a resurgence of COVID-19 infections that spanned the months of December 2020 and January 2021 (Fig. S1A in the supplemental material). Within a week of initiating the program, restrictions were minimally relaxed as the region progressed to the province of Ontario’s red (control) category, the second most restrictive category of the province’s COVID-19 response framework.

**FIG 1:**
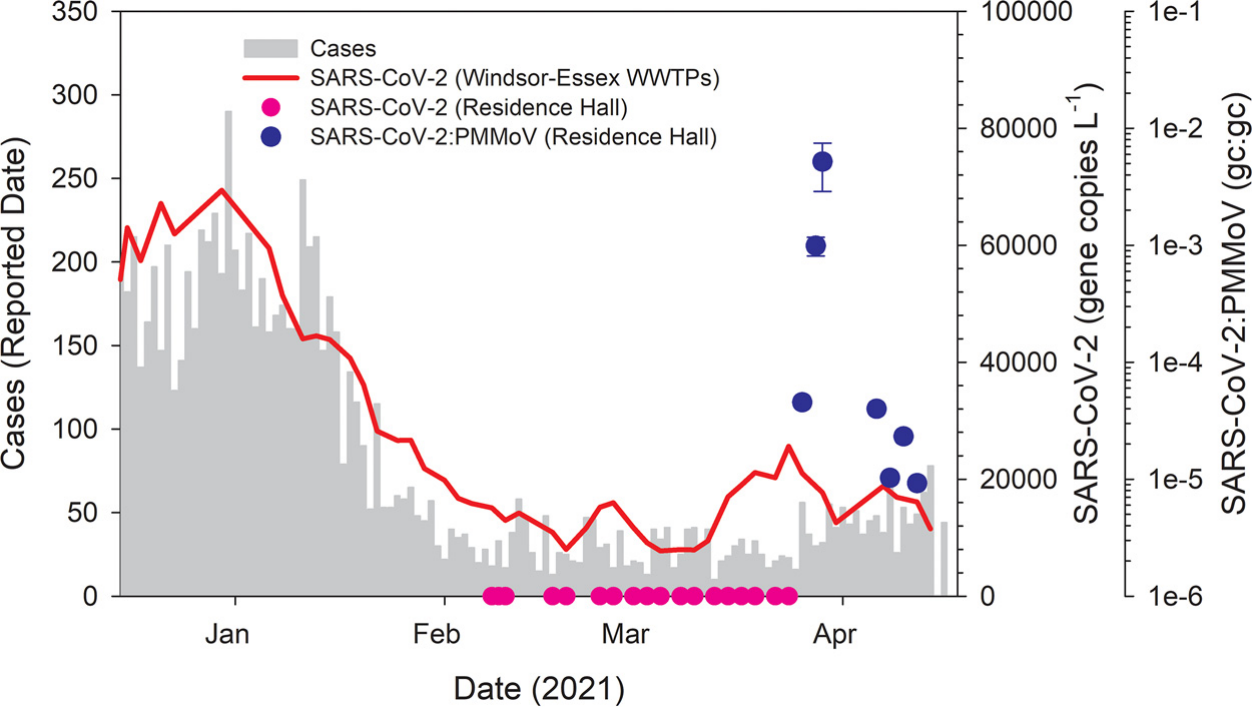
Concentration of the SARS-CoV-2 N1 gene target in wastewater superimposed onto COVID-19 cases in the Windsor-Essex region, plotted as a histogram. The N1 gene concentration is a 7-day running average of aggregate data from five WWTPs in Windsor-Essex, with the data weighted by population served (red line). These data are publicly available on a dashboard updated weekly (44). Sampling of residence hall wastewater by grab samples over 7 weeks yielded no detections of SARS-CoV-2 (pink circles). Following deployment of passive samplers, SARS-CoV-2 was detected in the residence hall sewer, plotted as the ratio of gene copies (gc) of SARS-CoV-2:PMMoV (blue circles; ±standard error [SE] where replicate samples were analyzed).

**FIG 2:**
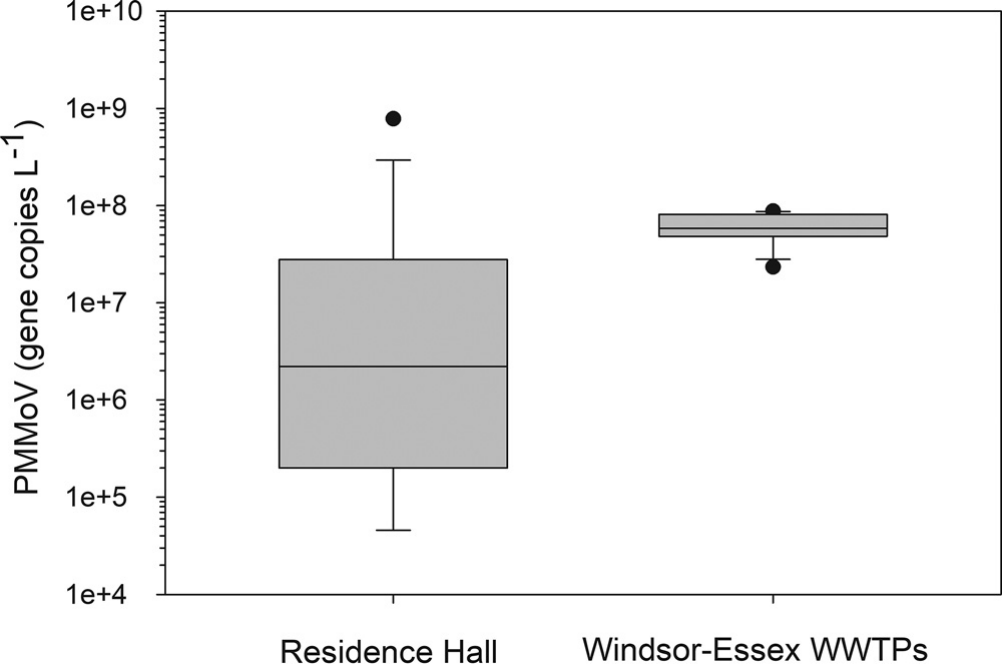
Concentration of PMMoV in wastewater from a university residence hall and as aggregate data across five WWTPs in the Windsor-Essex region. The data are presented as box and whisker plots showing the median gene concentrations. Vertical boxes around each median show the upper and lower quartiles, whereas whiskers extend from the 10th to 90th percentile. Potential outliers are shown as discrete points.

From early February through late March 2021, wastewater originating from a campus residence hall was collected as grab samples 3 times per week. Over this period, there was no detection of SARS-CoV-2 in the residence hall wastewater stream (Fig. 1). Over this same period, the concentration of SARS-CoV-2 in Windsor-Essex wastewater had stabilized at a low but detectable level following the December to January resurgence of infections, as demonstrated from surveillance of five wastewater treatment plants (WWTPs) (Fig. 1 and S1B). The comparability of data obtained from grab samples with 24-h composite samples obtained by autosampler has been investigated as part of several SARS-CoV-2 wastewater surveillance programs (34–36). While general agreement between the approaches has been reported, groups report within-day variability in terms of detection of SARS-CoV-2 using the grab sample approach, a concern that is magnified when dealing with a congregate living facility housing a small population such as a university residence hall. This concern was reflected by the variability in the concentration of *Pepper mild mottle virus* (PMMoV) yielded by grab samples, which ranged across 4 orders of magnitude at the residence hall, yielding a coefficient of variation (CV) of 2.83 (Fig. 2). In contrast, the concentration of PMMoV from 24-h composite samples averaged across five WWTPs in Windsor-Essex over the same time period showed only modest variation, yielding a CV an order of magnitude lower, at 0.38 (Fig. 2). While the increased variability in PMMoV at the residence hall was likely attributable to variability in the fecal contributions to the sewer, we cannot discount diet as a factor, especially considering the relatively small population accommodated at the residence hall.

To obtain a sample more representative of the residence hall community and their defecation patterns (37), a decision was made to implement the use of passive samplers from late March onwards. Unlike grab samples, which represent a point-in-time “snapshot,” passive samplers offer the advantage of providing a time-integrated measure of the sampled matrix and potentially greater sensitivity of viral detection, owing to the larger volume of sewage passing over the sampler compared to that which can be collected by a discrete sampling approach. Indeed, recent comparison of grab samples with Moore swabs targeting wastewater originating from a hospital admitting COVID-19 patients demonstrated that passive samplers can detect SARS-CoV-2 more consistently in wastewater (21). Likewise, Moore swabs provide data comparable to an autosampler, and both methods outperform grab samples to detect SARS-CoV-2 from municipal sewer access points (38). Arguments against adopting the use of passive samplers include challenges to quantifying viral concentrations with confidence. There are likewise uncertainties concerning the collection efficiency of the sampler, such as what fraction of virus passing across the sampler is retained and whether the virus retained by the sampler is subject to degradation over the collection interval.

Moore swabs yielded eluted concentrations of PMMoV that ranged over 2 orders of magnitude and a CV of 1.18, thus offering improved consistency over grab samples based on this metric. SARS-CoV-2 was detected in wastewater eluted from Moore swabs later the same week after passive sampling was first implemented (Fig. 1). The initial positive result triggered higher frequency sampling such that passive samplers were again deployed within 2 days following the initial positive and then daily over the next week. Testing resulted in the detection of SARS-CoV-2 in the residence hall wastewater during the initial 2 days of daily sampling, following which the virus was no longer detected through the end of the week. During the initial 4-day period in which SARS-CoV-2 was detected, the signal continued to increase in intensity, reflected by cycle threshold (*C_T_*) values for the N1 gene target as high as ∼27. Normalizing to the fecal indicator PMMoV eluted from the same passive sampler, the ratio increased by 3 orders of magnitude over this 4-day period (Fig. 1). Following 4 days of negative tests, sampling resumed following a holiday weekend, when SARS-CoV-2 was again detected in the residence hall wastewater, although with a signal intensity as normalized to PMMoV declining each day over a 7-day period.

At the time of the campus surveillance program, there was mounting concern globally over the emergence of SARS-CoV-2 VOCs. In North America, concern was focused largely on the Alpha VOC, which was reported to be more transmissible than the wild-type Wuhan strain (39, 40). Of particular concern to public health was a reported shift in the demographic of those infected trending to younger adults (40). Wastewater samples originating from the university residence hall were queried with an allele-specific primer extension reverse transcriptase quantitative PCR (RT-qPCR) assay targeting a D3L mutation on the SARS-CoV-2 N gene that is diagnostic for Alpha (41). These samples tested positive for the Alpha VOC coincident with the initial detection of SARS-CoV-2 in residence hall wastewater in late March and again in early April, following the reappearance of SARS-CoV-2 in the residence hall wastewater. While Alpha had become the dominant lineage of SARS-CoV-2 in parts of Ontario, especially in the greater Toronto area by mid-March (42), the Windsor-Essex region had reported just 25 cumulative cases (of 758 total reported cases) in March assigned to Alpha through to the time the VOC was detected in the residence hall wastewater in late March (43). Application of the allele-specific primer extension RT-qPCR assay to wastewater from Windsor-Essex showed no evidence of the Alpha VOC in the region through mid-March (Fig. S1B in the supplemental material), consistent with no more than 2 cases attributed to Alpha reported in the region per day through our initial detection of SARS-CoV-2 in the residence hall waste stream (43). By late March, this lineage had emerged, with Windsor-Essex wastewater yielding a weighted mean of 34% Alpha VOC, which increased to ∼60% through weekly testing into early June (44) (Fig. S1B in the supplemental material). This Alpha signal rose in parallel with locations elsewhere in the province, albeit staggered in onset. The rapid increase in the dominance of the Alpha VOC in the wastewater was mirrored by clinical data, with the region logging >1,000 cumulative COVID-19 cases attributed to Alpha by early May (45).

### Insights into fecal shedding rates from wastewater surveillance.

Since initial reports showing that SARS-CoV-2 can be detected in wastewater, a promising, albeit somewhat elusive, extension of WBE has been its use for estimating community infections within a sewer catchment (7, 9, 19, 34, 46). The uncertainties related to estimating absolute numbers of community infections are numerous and continue to be a challenge to realizing this application of WBE. Highlighting these uncertainties is our lack of understanding of fecal shedding rates, as well as stability of the virus within the sewershed (12), where viral particles may be entrained from several hours to as long as ∼2 days, depending on the sewer network (47).

Where the prevalence of COVID-19 infections has been estimated from wastewater data, estimates of fecal shedding rates to derive the loading of SARS-CoV-2 into wastewater have largely invoked data from a limited number of clinical studies examining excretion of the virus in human feces. In these studies, the viral titer has been estimated to differ by several orders of magnitude (48, 49). Likewise, uncertainty surrounds the ubiquity and duration of fecal shedding. A systematic review and meta-analysis of 13 studies examining SARS-CoV-2 in stool showed a mean shedding duration of 17.2 days, with a maximum duration of 126 days (50). Further, VOCs may exhibit different shedding patterns than the wild-type strain, with recent studies providing evidence for a higher viral load (51) and prolonged shedding time in the upper respiratory tract of patients infected with the Alpha lineage (52, 53).

WBE programs associated with congregate living facilities offer a unique opportunity to calculate fecal shedding rates from a defined community. By combining wastewater surveillance with clinical data derived from testing individuals housed at these facilities, it is possible to extrapolate an approximate fecal shedding rate. Further, considering that sample collection points are typically adjacent to the facilities being tested, virus contributions to the wastewater stream are presumably recent, thus negating some of the concerns over factors affecting the stability of SARS-CoV-2 in sewers. Aggregating wastewater and clinical data, the WBE program at the University of Arizona yielded among the first estimates of fecal shedding rates for SARS-CoV-2 from a nonclinical setting (32). In their study, implemented over a 3-month period in fall 2020 and covering 13 dormitories, 81 wastewater samples tested positive for SARS-CoV-2 and triggered the clinical testing of students living in dorms, resulting in diagnoses of 711 cases of COVID-19, of which 79.2% were classified as asymptomatic. Because infected students were relocated to quarantine facilities that did not contribute to the study’s sewershed, infections associated with the dormitories were considered incident infections. Aggregating the data from all the dorms yielded a mean SARS-CoV-2 shedding rate of 6.84 ± 0.77 log_10_ gene copies (gc)/g feces, based on the N1 gene (32).

The case study presented here, based on the experience of implementing WBE at the University of Windsor, likewise offered a unique opportunity to estimate fecal shedding rates attributed to a defined community, but with a signal that was easier to interpret than in similar studies elsewhere. In this case, there was but a single occupied residence hall, having had no detection of SARS-CoV-2 in the wastewater over the 7 weeks leading up to the initial detection, which triggered clinical testing of the building occupants. Two individuals tested positive for COVID-19, with both individuals relocated to a quarantine facility on campus less than 2 days after the wastewater data were reported to campus administration. Analysis of the wastewater showed that it was positive for the Alpha VOC. Limiting our analysis to just the 4-day period encompassing the initial detection of SARS-CoV-2 in the residence hall wastewater, through to the clinical testing and quarantine of the two individuals who tested positive (Fig. 1), we report fecal shedding rates progressing in their intensity and ranging across 3 orders of magnitude, from 3.93 log_10_ gc/g feces to 5.99 log_10_ gc/g feces, based on the N1 gene target. These rates are lower than those reported from the Arizona study and must be interpreted with some caution, given the uncertainties surrounding fecal shedding, including reports that some infected individuals do not shed SARS-CoV-2 in their feces (51). One must also consider the distinction that the cases presented here were specific to the Alpha VOC. Finally, we recognize that our estimates of fecal shedding rates are based on indirect assessment derived from ratios of SARS-CoV-2:PMMoV obtained from passive samplers and using flow rates indirectly estimated from building water usage.

Despite the uncertainty in estimating the fecal shedding rates in this study, the linear progression in intensity of shedding over 4 days, as shown by the SARS-CoV-2:PMMoV ratio, was consistent with recent reports showing an estimated time from the onset of shedding to peak viral load of 4.31 days (51) to around 6 days (54). While we do not know if the subjects reached peak viral load by the time they were relocated to quarantine, the rapid increase in shedding intensity of more than 2,000% as infection progressed between days 3 and 4 would suggest that the peak was being approached.

Unfortunately, as sampling resumed following a holiday weekend and SARS-CoV-2 was once again detected in the residence hall wastewater, data interpretation was complicated due to the return of the students who had previously been quarantined. Thus, while a new infection was most likely responsible for the reemergent SARS-CoV-2 signal in wastewater detected in early April, that individual was removed to quarantine the following day, and the 60% decline in signal that followed was attributed to reduced rates of shedding by the students deemed no longer to be infectious, but still classified as convalescent. A lack of detection of SARS-CoV-2 by mid-April corresponded with day 19 of the onset of infection associated with the initial subjects and thus close to the 17.2 day mean shedding duration reported previously (50).

### Public health response and clinical confirmation of infection.

Detection of SARS-CoV-2 in the residence hall wastewater sample triggered a rapid public health response by the University of Windsor COVID-19 Case Response Team and the Windsor-Essex County Health Unit (WECHU). Results from the initial detection of SARS-CoV-2 in residence hall wastewater were available by 12:00 the day following the Moore swab retrieval and communicated to university administration by 17:00. The university administration requested clarification regarding the data and conferred with Health and Safety at the university, at which time the concern was elevated, and WECHU was contacted by 20:00. Shortly thereafter, student residents and employees whose duties included access to the residence hall were sent electronic notification of the likelihood of a positive SARS-CoV-2 case within the facility and were encouraged to self-isolate. They were also apprised of a clinical testing unit to be mobilized to the residence hall the following morning. Over the following 2 days, over 195 nasopharyngeal swabs were collected by a mobile testing team, with test results communicated within 24 h. From the initial cohort tested, a single positive SARS-CoV-2 infection was confirmed. This individual, along with a close contact who was also a resident of the facility, were moved to a separate quarantine facility on campus by midday the following day once the initial test results were obtained. This close contact also tested positive as a part of testing of the second cohort 1 day later. RT-qPCR assay of the clinical samples for these individuals yielded *C_T_* values of ≤35, which triggered subsequent screening for the diagnostic N501Y and E484K VOC mutations associated with the spike (S) gene using a multiplex RT-qPCR assay (55). Samples from both individuals were positive for the N501Y mutation and negative for E484K, and the individuals presumed to be infected with the Alpha VOC based on Public Health Ontario criteria (55). This clinical diagnosis was consistent with wastewater testing, which identified Alpha by targeting the diagnostic D3L mutation on the N gene.

All other clinical tests performed following the initial detection of SARS-CoV-2 in the residence hall wastewater were negative. Quarantine of the two Alpha-infected individuals had immediate implications for wastewater screening, with a return to nondetection of SAR-CoV-2 during daily surveillance in the days following. With the resumption of testing following a holiday weekend yielding a positive result for SARS-CoV-2, the University COVID-19 Case Response Team was again notified, and we learned that a third student resident of the facility had been confirmed positive for COVID-19 earlier in the day, after the student had sought testing the day prior. Upon learning of the test result, this third individual was relocated to the quarantine facility. Wastewater testing resumed the following day, with another positive result suggesting that there remained one or more individuals at the residence hall actively shedding virus. Again, this result triggered a public health response, and students were notified of a previously scheduled university-coordinated testing clinic on campus, which attracted 65 student residents for testing, all of whom tested negative. We subsequently learned that the two residents who were initially quarantined had been approved to return to the residence hall after 10 days had elapsed, as they were deemed no longer infectious. This information combined with the knowledge that all student residents who chose to be tested following the reemergence of SARS-CoV-2 in residence hall wastewater were negative suggests that convalescent shedding of SARS-CoV-2 was likely responsible for the virus persisting in the wastewater stream through mid-April.

## CONCLUSIONS

The University of Windsor has implemented a multipronged surveillance-based informative framework that combines wastewater testing with voluntary pooled saliva-based RT-qPCR screening to monitor for SARS-CoV-2 as part of a return to campus strategy (44) that will continue into the fall 2021 semester. Here, we report on the wastewater-testing component of this screening program and demonstrate that a WBE program targeting a congregate living facility on a university campus can lead to actionable responses by the university administration and public health, having the potential to avert an outbreak of COVID-19. As the COVID-19 pandemic unfolds, wastewater surveillance continues to be refined and informed by our growing understanding of SARS-CoV-2, the emergence of variants, and the persistence of the virus in wastewater. Actioning this emerging discipline of WBE into a public health response requires buy-in from administrators and public health authorities, whose confidence is often gained slowly. However, as with much of the decision-making associated with the pandemic, decisions have had to be made quickly without the benefit of having all evidence in place. In this case, growing confidence in this evolving discipline resulted in the rapid deployment of a mobile testing team, who identified the infected individuals and relocated them to quarantine to avert an outbreak.

This work highlighted some of the challenges faced by WBE in congregate living facilities, including unpredictable wastewater flow and the confounding effect of convalescent shedding on interpreting the SARS-CoV-2 signal in wastewater. While many universities proactively relocate infected students into quarantine facilities, these are not meant to be long-term displacements. Students who are deemed no longer infectious but are still classified as convalescing are normally approved to return to their assigned accommodations. Convalescent shedding of SARS-CoV-2 can persist for weeks to months; thus, universities planning to use WBE need to consider this as part of their quarantine plans (25).

As the current pandemic winds down, with vaccination efforts ramping up globally, the longer-term application of WBE on campuses and elsewhere will need to be considered (26): A recent commentary argued that wastewater surveillance can “have a second act” to inform vaccine uptake, especially if applied upstream within a sewershed to target neighborhoods or congregate living facilities (56). These data would inform a public health strategy to encourage and facilitate the vaccination of residents in these areas. Likewise, the value of WBE beyond the current pandemic is increasingly being recognized, and there are calls to establish national wastewater surveillance systems having applications for detecting other well-known disease agents, including foodborne pathogens shed in feces, or to be applied to new pandemics caused by emerging pathogens (57). Such calls to action have been prompted by the rapid evolution of this public health tool as applied to COVID-19 and the realization that WBE represents a “community swab” which is both cost-effective and scalable.

## MATERIALS AND METHODS

### Sample collection and location.

The University of Windsor is a comprehensive public research university located in southwestern Ontario on the Canada-U.S. border enrolling >16,000 students. As with postsecondary institutions across Canada, the university transitioned to remote learning for the 2020 to 2021 academic year. This reduced the footprint of students and employees on campus by ∼70% and meant that those students requiring on-campus accommodations could be housed in a single residence hall, with a second on-campus residence used as necessary as a quarantine facility. Sampling of the residence hall on the campus of the university was initiated in early February 2021, targeting a sewer line originating from the residence which empties into the municipal sewer system for the city of Windsor, Ontario. During the spring semester 2021, the residence hall housed 186 students living in 2-bedroom suites, with each suite sharing a common toilet facility. The residence hall contains two wings, each with separate sewer lines. The sewer line chosen for sampling serviced the north wing of the building, which housed 86 students. From early February through late March, wastewater was collected as grab samples 3 times each week, usually between 10:00 and 11:00 local time, using polypropylene bottles, with successive grab samples consolidated to fill a 250-ml bottle.

Beginning in late March, a passive sampler approach was adopted, with the use of a modified Moore swab (21, 24, 58). Briefly, this approach used a feminine hygiene product (tampon) connected by fishing line to a magnetic carabiner attached to the inside rim of the sewer cover. The modified Moore swabs were deployed in duplicate into the wastewater stream, where they resided for ∼20 h prior to retrieval. Deployment lasted from midafternoon through late morning the following day. Once retrieved, the swabs were collected into sealable plastic bags and transported in a cooler to the laboratory for processing. The time elapsed between sample collection and their receipt in the laboratory was no longer than 30 min.

### Sample processing.

The sample volume processed varied depending on the solids content and ranged between 12 and 105 ml (median, 55 ml). Grab samples of raw wastewater were mixed by gentle shaking, and a particle-associated fraction was concentrated by filtration through 0.22-μm Sterivex cartridge filters (MilliporeSigma, Burlington, MA), followed by flash-freezing the filter in liquid nitrogen as described previously (59). The filtrate was collected into a sterile 50-ml centrifuge tube, followed by the addition of 0.05% (vol/vol) Tween 20, and concentrated using a concentrating pipette (CP) Select (InnovaPrep, Drexel, MO) with ultrafiltration PS hollow fiber concentrating tips following a custom protocol (60). Using the CP Select, the filtrate was concentrated to ∼300 μl and then flash-frozen in liquid nitrogen.

Upon transition to the use of passive samplers, individual Moore swabs were placed into the barrel of a disposable 50-ml syringe, and the liquid retained in the swab was plunged into a sterile 50-ml centrifuge tube. The filtrate was concentrated by ultrafiltration as described above using the CP Select. RNA was extracted from the filters and the concentrated filtrate following the manufacturer’s instructions using the AllPrep PowerViral DNA/RNA kit (Qiagen, Germantown, MD). The samples were not treated with DNase upon extraction.

### RT-qPCR.

Assays for SARS-CoV-2 targeted regions of the nucleocapsid (N) gene using U.S. Centers for Disease Control and Prevention (CDC) primers and probes for the N1 region (61). The Alpha VOC assay targeted a region of the N gene containing D3L, a signature mutation diagnostic of Alpha (41). The *Pepper mild mottle virus* (PMMoV), which like SARS-CoV-2 is a positive-sense, single-stranded RNA virus, was selected as a fecal indicator and quantified using primers and probes described previously (62).

Reactions contained 5 μl of RNA template mixed with 10 μl of 2× RT-qPCR master mix (Takyon dry one-step RT probe master mix no ROX; Eurogentec, Liège, Belgium) and primers and probes in a final reaction volume of 20 μl. Due to repeated incidence of inhibition with wastewater samples, the template was diluted 1:5 in all reactions. Technical triplicates were run for detection of gene targets. Thermal cycling was performed using an MA6000 qPCR thermocycler (Sansure Biotech, Changsha, China). RT was performed at 48°C for 10 min, followed by polymerase activation at 95°C for 3 min, 50 cycles of denaturation, and annealing/extension at 95°C for 10 sec and 60°C for 45 sec. The EDX SARS-CoV-2 synthetic RNA standard (Exact Diagnostics, Fort Worth, TX, USA) was used to create a 5-point standard curve to quantify the N1 gene target, whereas synthetic RNA containing the D3L mutation (AR-S SARS-CoV-2 RNA control 14; Twist Bioscience, South San Francisco, CA) served as a positive control for Alpha. For PMMoV, a sample pooled from multiple WWTPs in southwest Ontario and quantified by RT-Droplet digital PCR was used to generate standard curves (47). No template controls yielded no amplification, and we report a limit of detection of 5 gene copies of N1 per reaction (≥95% probability of detection).

### Estimating fecal shedding rates.

The approach described as part of the WBE program at the University of Arizona (32) was adopted to estimate the fecal shedding rates with a few modifications. This approach combines data from wastewater surveillance and clinical testing targeting SARS-CoV-2, along with estimates of flow rates during the period when the passive samplers were deployed. Fecal shedding rates in units of gene copies per gram feces (gc/g feces) were estimated using the following equation:

(1) $$FS=\frac{\left(\text{VC}\times \text{Q}\times \text{h}\right)}{\left(\text{G}\times \text{I}\right)}$$

where *VC* is the N1 gene concentration in units of gene copies per liter, *Q* is the flow rate of wastewater leaving the residence hall in units of liters per minute, and *h* is a conversion factor for time changing minutes to days. In the denominator, *G* represents the median per capita wet weight mass of feces from high-income countries (126 g/person/day [63]) and *I* is the total number of infected persons potentially contributing to the SARS-CoV-2 signal in wastewater.

Given the challenges of determining the absolute concentration of the target genes using a passive sampler (64), VC was calculated from the ratio of SARS-CoV-2:PMMoV eluted from passive samplers and using the median PMMoV gene concentration determined from 17 grab samples collected over a 7-week period spanning February and March (Fig. 2). PMMoV is widely used as a fecal indicator to normalize SARS-CoV-2 in wastewater (19, 47). Determining the flow rates was likewise challenging, given the small population serviced, which generated intermittent flow through the sewers. Others have attempted direct measures of flow; however, problems exist using conventional flow meters, especially when the flow is very low and the sensors are not completely immersed in water (32). As an alternative, water consumption as measured by a public utilities meter in the building was used to estimate the wastewater flow. Given that the water supplied to the student residence is used entirely within the building, the resulting wastewater flow discharged into the sewer system is expected to be very similar (65). Inflow and infiltration were considered negligible due to the short path (<20 m) of the sewer lateral from the residence hall to the main sewer line. Water consumption was normalized to the number of students resident in the wing of the residence hall that was monitored and adjusted to better estimate the percent water use during the normal time of deployment of the passive samplers.

### Ethics review.

Ethics consultation was sought from the University of Windsor’s research ethics board, and both the wastewater project and the information on the cases described were considered exempt from ethics review under the Canadian *Tri-Council Policy Statement: Ethical Conduct for Research Involving Humans—TCPS 2* (2018), articles 2.4 and 2.5. The Windsor-Essex County Health Unit reviewed the study and verified that no personal health information was involved in the analysis or summary.
