# Supplementary material for: Averting an Outbreak of SARS-CoV-2 in a University Residence Hall through Wastewater Surveillance
Source: Microbiol Spectr. 2021 Oct 6;9(2):e00792-21. doi: 10.1128/Spectrum.00792-21 (PMC8510253; doi:10.1128/Spectrum.00792-21)
Supplement: SUPPLEMENTAL FILE 1 — Supplemental material. Download SPECTRUM00792-21_Supp_1_seq6.pdf, PDF file, 0.2 MB [file spectrum00792-21_supp_1_seq6.pdf]

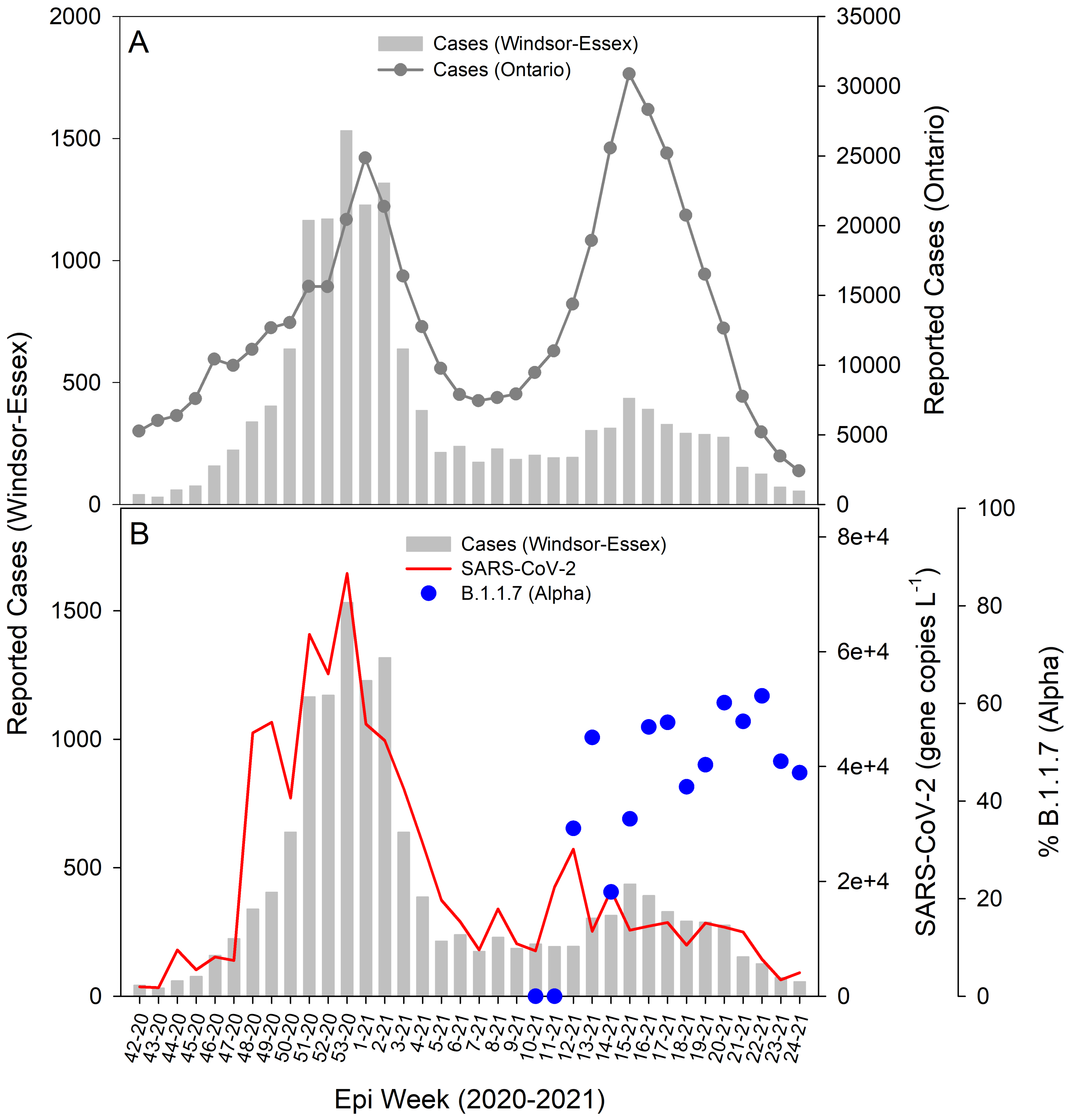

A
Cases (Windsor-Essex)
Cases (Ontario)
B
Cases (Windsor-Essex)
SARS-CoV-2
B.1.1.7 (Alpha)
Reported Cases (Windsor-Essex)
Reported Cases (Ontario)
SARS-CoV-2 (gene copies L-1)
% B.1.1.7 (Alpha)
Epi Week (2020-2021)
42-20 43-20 44-20 45-20 46-20 47-20 48-20 49-20 50-20 51-20 52-20 53-20 1-21 2-21 3-21 4-21 5-21 6-21 7-21 8-21 9-21 10-21 11-21 12-21 13-21 14-21 15-21 16-21 17-21 18-21 19-21 20-21 21-21 22-21 23-21 24-21
0 500 1000 1500 2000
0 5000 10000 15000 20000 25000 30000 35000
0 2e+4 4e+4 6e+4 8e+4
0 20 40 60 80 100

Figure S1. A) Cases reported by epidemiological (epi) week for Windsor-Essex (grey bars) and the Province of Ontario (grey circles). The Windsor-Essex region mirrored the Province in severity associated with the COVID-19 resurgence that spanned late November 2020 – January 2021 (epi weeks 40 [2020] – 4 [2021]) accounting for up to 8% of all cases despite representing only ~3% of the population of the Province. While Windsor-Essex experienced a resurgence of COVID-19 cases fueled by the Alpha variant of concern (VOC) between late March through May (epi weeks 13-20), it was less pronounced than throughout the Province as a whole accounting for only 1-2% of all reported cases. B) Concentration of SARS-CoV-2 N1 gene target in wastewater superimposed on COVID-19 cases reported by epi week in the Windsor-Essex region (grey bars). N1 gene concentration is a 7-day running average of aggregate data from five WWTPs in Windsor-Essex with data weighted by population served (red line). These five WWTPs treat wastewater accounting for 85% of the regions population. As part of the Ontario Wastewater Surveillance Initiative, 24-h composite samples from these plants are collected at a frequency of up to thrice weekly. Samples were processed the same day as collection as described for grab samples from residence hall wastewater in the Materials and Methods. Occurrence of the Alpha VOC was determined by N-gene primer extension assay targeting the D3L mutation as described in the Materials and Methods. An approximation of Alpha VOC as a proportion of total SARS-CoV-2 (blue circles) was determined by RT-qPCR performed in parallel using primers and probes for D3L and the CDC N1 gene target quantified using the same synthetic RNA standard (AR-S SARS-CoV-2 RNA Control 14; Twist Bioscience, South San Francisco, CA) with the N1 value serving as the denominator. RT-qPCR assays for the Alpha VOC were carried out 1-2 × weekly on a subset of the Windsor-Essex region WWTPs collectively accounting for 75% of the regions population. The Alpha variant emerged in

Windsor-Essex in late March, 2021 and became the dominant lineage circulating in the region accounting for ~60% of all SARS-CoV-2 variants by early June.
